# The value of stereotactic biopsy of primary and recurrent brain metastases in the era of precision medicine

**DOI:** 10.3389/fonc.2022.1014711

**Published:** 2022-12-20

**Authors:** Sophie Katzendobler, Anna Do, Jonathan Weller, Kai Rejeski, Mario M. Dorostkar, Nathalie L. Albert, Robert Forbrig, Maximilian Niyazi, Rupert Egensperger, Joerg-Christian Tonn, Louisa von Baumgarten, Stefanie Quach, Niklas Thon

**Affiliations:** ^1^ Department of Neurosurgery, University Hospital, Ludwig-Maximilians-Universität (LMU) Munich, Munich, Germany; ^2^ Department of Medicine III, Hematology and Oncology, University Hospital, Ludwig-Maximilians-Universität (LMU) Munich, Munich, Germany; ^3^ German Cancer Consortium (DKTK), Partner Site Munich, German Cancer Research Center (DKFZ), Heidelberg, Germany; ^4^ Center for Neuropathology and Prion Research, Ludwig-Maximilians-Universität (LMU) Munich, Munich, Germany; ^5^ Department of Nuclear Medicine, University Hospital, Ludwig-Maximilians-Universität (LMU) Munich, Munich, Germany; ^6^ Institute of Neuroradiology, University Hospital, Ludwig-Maximilians-Universität (LMU) Munich, Munich, Germany; ^7^ Department of Radiation Oncology, University Hospital, Ludwig-Maximilians-Universität (LMU) Munich, Munich, Germany

**Keywords:** stereotactic biopsy, brain metastases, recurrent brain metastases, pseudoprogression, precision medicine, molecular diagnostics, image guided procedures, targeted therapy

## Abstract

**Background:**

Brain metastases (BM) represent the most frequent intracranial tumors with increasing incidence. Many primary tumors are currently treated in protocols that incorporate targeted therapies either upfront or for progressive metastatic disease. Hence, molecular markers are gaining increasing importance in the diagnostic framework of BM. In cases with diagnostic uncertainty, both in newly diagnosed or recurrent BM, stereotactic biopsy serves as an alternative to microsurgical resection particularly whenever resection is not deemed to be safe or feasible. This retrospective study aimed to analyze both diagnostic yield and safety of an image-guided frame based stereotactic biopsy technique (STX).

**Material and methods:**

Our institutional neurosurgical data base was searched for any surgical procedure for suspected brain metastases between January 2016 and March 2021. Of these, only patients with STX were included. Clinical parameters, procedural complications, and tissue histology and concomitant molecular signature were assessed.

**Results:**

Overall, 467 patients were identified including 234 (50%) with STX. Median age at biopsy was 64 years (range 29 – 87 years). MRI was used for frame-based trajectory planning in every case with additional PET-guidance in 38 cases (16%). In total, serial tumor probes provided a definite diagnosis in 230 procedures (98%). In 4 cases (1.7%), the pathological tissue did not allow a definitive neuropathological diagnosis. 24 cases had to be excluded due to non-metastatic histology, leaving 206 cases for further analyses. 114 patients (49%) exhibited newly diagnosed BM, while 46 patients (20%) displayed progressive BM. Pseudoprogression was seen in 46 patients, a median of 12 months after prior therapy. Pseudoprogression was always confirmed by clinical course. Metastatic tissue was found most frequently from lung cancer (40%), followed by breast cancer (9%), and malignant melanoma (7%). Other entities included gastrointestinal cancer, squamous cell cancer, renal cell carcinoma, and thyroid cancer, respectively. In 9 cases (4%), the tumor origin could not be identified (cancer of unknown primary). Molecular genetic analyses were successful in 137 out of 144 analyzed cases (95%). Additional next-generation sequencing revealed conclusive results in 12/18 (67%) cases. Relevant peri-procedural complications were observed in 5 cases (2.4%), which were all transient. No permanent morbidity or mortality was noted.

**Conclusion:**

In patients with BM, frame-based stereotactic biopsy constitutes a safe procedure with a high diagnostic yield. Importantly, this extended to discerning pseudoprogression from tumor relapse after prior therapy. Thus, comprehensive molecular characterization based on minimal-invasive stereotactic biopsies lays the foundation for precision medicine approaches in the treatment of primary and recurrent BM.

## Introduction

Brain metastases (BM) occur in up to 40% of all patients with solid tumors over the course of disease (1, 2). Patients suffering from lung carcinoma, both non-small cell lung cancer (NSCLC) and small cell lung cancer (SCLC), as well as breast cancer and malignant melanoma are most commonly affected (1–3). Due to a short median survival time of less than 12 months across nearly all primary sites and the often-limited efficacy of systemic therapy, clinical management of BMs can be exhausting and requires multidisciplinary expertise (1, 2). According to the 2021 joint European Association of Neuro-Oncology (EANO) and European Society for Medical Oncology (ESMO) guidelines for diagnosis, treatment and follow-up of patients with brain metastasis from solid tumors, any new neurological deficit in a cancer patient should always be suggestive of BM (4). Suspicious brain lesions may also appear on routine check-up magnetic resonance imaging (MRI)-scans of cancer patients, incidentally or during the recommended work-up (2). Singular lesions amenable to safe surgical resection should be operated upon, space-occupying lesions may even require urgent decompression (4, 5). Microsurgical tumor resection serves both therapeutic and diagnostic purposes, but at the risk of potential surgical complications particularly in frail patients (6).

Versatile histopathological and molecular-genetic analyses, however, should also be available in all unclear cases with multiple or highly eloquent lesions, particularly in patients with a history of more than one primary tumor, and those with unclear tumor status after therapy (7–9). Novel high-throughput sequencing methods have improved our understanding of individual cellular and molecular tumor targets. As a result, multiple novel personalized treatment strategies have been identified to treat cancer patients, thus opening novel treatment options for BMs. For example, in patients with Her2-positive breast cancer BMs (10, 11), those with ALK-rearranged (12, 13) or EGFR-mutated (14, 15) NSCLC BMs, and for BRAF V600 E mutated melanoma BMs (16), targeted therapies with significant intracranial activity are available. Still, there may be discrepancies between the actionable mutations of the primary tumor and their respective BM (17) and thus tissue-based analyses of BM can be necessary to guide therapy.

Due to the high recurrence rate of BM, follow-up imaging with short intervals is pivotal to monitor the course of disease and to potentially re-adjust therapy in case of tumor progression. However, suspicious lesions on MRI-scans can also be a manifestation of post-therapeutic changes, e.g., tissue necrosis after a radiation procedure or inflammatory reactions during immunotherapies, also termed pseudoprogression (18, 19). Due to similar visual characteristics, correct differentiation from tumor recurrence can be a diagnostic challenge. The response assessment in neuro-oncology (RANO) working group recommends O-(2-^18^Fluorethyl)-L-tyrosine ([^18^F] FET PET) to discriminate true tumor progression from pseudoprogression (20–22). Nevertheless, in unclear cases tissue acquisition remains the gold standard to resolve this diagnostic quandary and to select the appropriate treatment modality (18, 23).

Consequently, minimally invasive biopsy techniques are of high importance in the field of brain metastases (4, 5). Even though stereotactic frame-based biopsy represents a well-established procedure, general analyses of BM biopsy cases and their respective histopathologic results have only been performed in a few studies to date. Importantly, these studies have mostly lacked in-depth molecular data and concomitant analyses of the associated risk profile. With the present study, we aim to delineate diagnostic accuracy, intervention-related risks and the diagnostic benefit of stereotactic biopsy for suspected BM.

## Materials and methods

### Study population

Our neurosurgical database was retrospectively searched for all patients undergoing any surgical procedure for suspected brain metastases between January 2016 and March 2021. Of these, only patients undergoing stereotactic biopsy were included. Ethical approval for this analysis was obtained from the ethics committee of the Ludwigs-Maximilians University Hospital (project number 22-0476). Patients provided informed written consent to allow for anonymous or pseudonymous data handling.

A standardized set of demographic, radiological, neuropathological, and clinical data was obtained. This included information on any known primary tumor as well as results of histological and, whenever conducted, molecular diagnosis. Complications were evaluated according to the Common Terminology Criteria for Adverse Events (CTCAE 5.0) classification system (24).

### Stereotactic biopsy technique

A highly standardized, frame-based, imaging-guided stereotactic biopsy technique was applied in all patients (23, 25).

Preoperative workup comprised a 1.5 or 3T MRI scan (with T2 and T1 sequences before and after application of a Gadolinium-based contrast agent and MR-angiography sequences) that was acquired one day prior to surgery and fused with an intraoperative, contrast-enhanced computed tomography (CT) angiography scan with the patients’ head fixed in the frame. If available, PET imaging data based on [^18^F] FET was included in the triplanar trajectory planning (Brainlab^®^ Elements Stereotactic Planning). At our center, [^18^F] FET-PET is used as an additional diagnostic examination method for BMs, primarily during the course of the disease in cases of suspected local recurrence after (radiation) therapy and to identify reactive changes (26, 27). The indication for [^18^F] FET-PET is consented for each individual patient within the interdisciplinary neuro-oncological tumor board.

Each trajectory was meticulously planned to harvest maximal active tumor tissue (no necrosis) and to avoid any risk of vascular damage, contact to sulci or cerebrospinal fluid (CSF) drainage, which may lead to intraoperative brain-shift with subsequent mismatch between planning MRI and real anatomy. A phantom frame was used to confirm correct 3-dimensional angulation prior to surgery in all patients. A skin incision of 4-6 millimeters (mm) was made and followed by a frame-guided burr hole trepanation with a diameter of 3 mm. After perforation of the dura through advancing a sharp trocar, a blunt trocar is used to reach the lesion. Subsequently, after inserting a rigid tube, multiple small tissue samples of 1 mm^3^ each were taken by utilizing a designated biopsy forceps inserted in the tube. An experienced neuropathologist was on site in the operating room (OR) during the procedure to examine whether the material obtained was sufficient in terms of quantity and quality for gaining a diagnosis. In our routine protocol, the first tissue samples are already used for smear preparation in order to limit the number of tissue samples taken that are necessary for a comprehensive neuropathologic diagnosis. Thereafter, the skin was closed with a suture. A routine control CT was performed within 24 hours to exclude hemorrhage and to confirm the correct site of tissue sampling in case of an inconclusive neuropathological finding.

### Neuropathological diagnosis and molecular genetic analyses

Histopathological and molecular diagnosis including next-generation sequencing was performed according to EANO guidelines at the Center for Neuropathology and Prion Research of the University Hospital Munich (28). To determine the origin of the respective BM, basic morphology is investigated in a first step to differentiate between carcinomas, lymphomas and melanomas. Immunohistochemical profiles of BM may be indicative of the site and lineage of the primary tumor. In case of a cerebral adenocarcinoma of unknown primary, TTF-1 status was investigated, as positive results are strongly associated with lung cancer and thyroid cancer. CK7 negativity and CK20 positivity were studied for potential evidence of colorectal cancer. Neuro-endocrine differentiation was tested using chromogranin, synaptophysin antibodies directed against specific hormones (e.g. insulin, gastrin, and glucagon). When sarcoma or other related mesenchymal primary malignancies were suspected, immunohistochemical panels for mesenchymal tumors were utilized (vimentin, desmin, S100) (28). In the absence of clear neuropathologic diagnostic criteria, when predominantly reactive changes were detected after tumor therapy without unequivocal tumor cell evidence, the neuropathologic presumption of a pseudoprogression was made, but this was always interpreted in light of the clinical course and imaging findings. This also includes the distinction from radiation necrosis, which was expressed if in particular necrosis zones and vascular proliferates were detected.

### Statistics

Patient-related, clinical and molecular information was collected and anonymized. Data analysis and descriptive statistics were performed using IBM SPSS Statistic software v25.0 (IBM, Armonk, New York, USA). When normal distribution of data sets was to be assumed, median and range were calculated. For comparison of absolute numbers, percentages were calculated. Subgroups were compared according to categorical and continuous variables. The level of significance was set at 0.05. The time between treatment and re-biopsy was compared between patients with true tumor progression or pseudoprogression using Log-rank test. Hazard Ratios (HR) were calculated and Confidence Intervals (CI) were given.

## Results

### Patients, procedure and tumor characteristics

**Figure 1:**
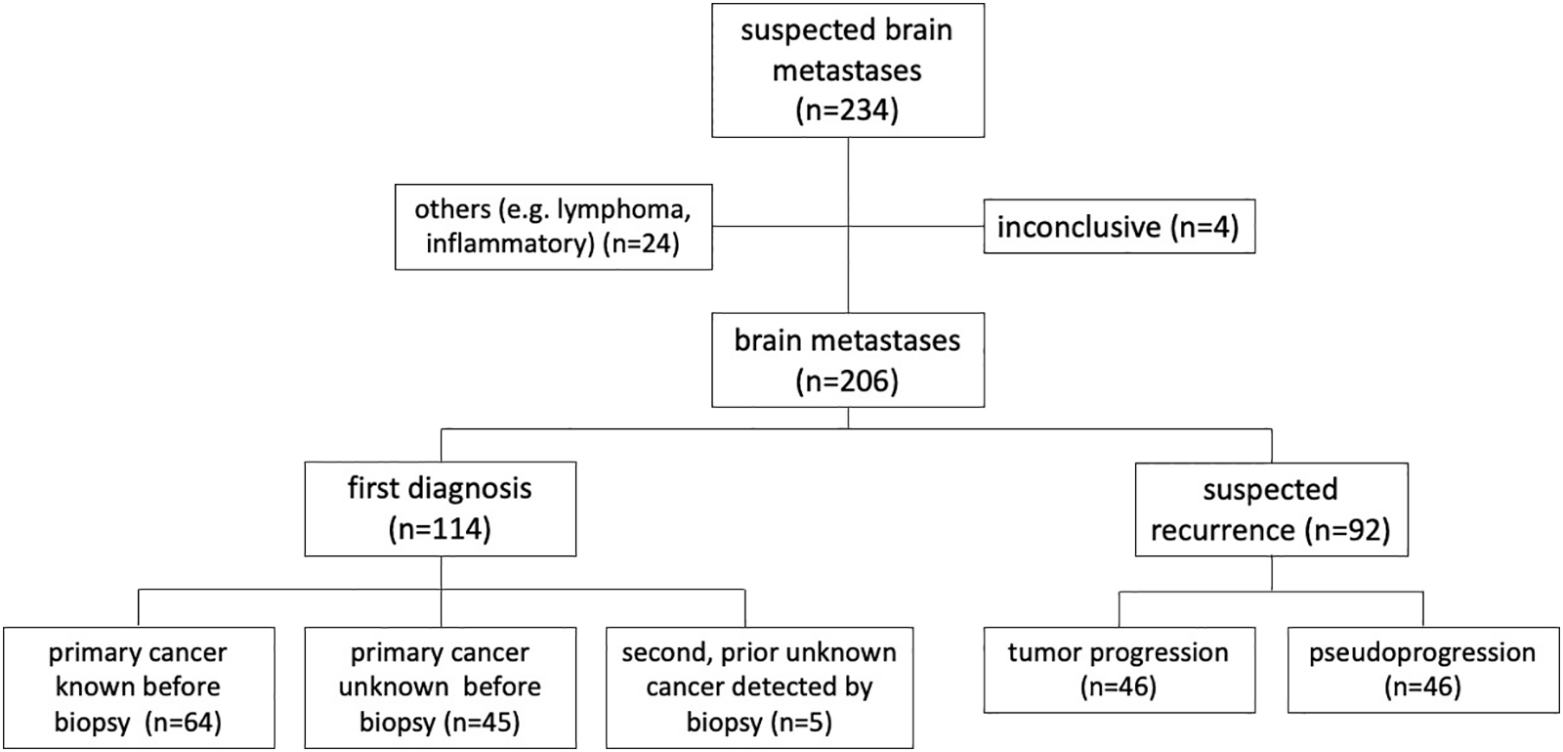
Study population.

Between January 2016 and March 2021, 467 patients underwent neurosurgical procedures for suspected BM with 234 (50%) stereotactic biopsies. Of the latter, 24 (12%) were excluded due to non-metastatic tissue (mainly cerebral lymphomas and inflammatory reactions). In 4 cases, histopathology and molecular analyses of lesional tissue samples was inconclusive, leaving a total number of 206 biopsied BM patients for further analyses (see **Figure 1**). In this study population, median age was 64 years, ranging from 29 to 87 years. 106 patients (52%) were female.

Out of 159 (77%) lesions with lobar location, 78 were left sided and 18 were located bilaterally. 39 BM (19%) were deep seated (insula, thalamus, pineal region, cerebellum) and 8 (3.9%) lesions involved the brainstem (**Table 1**).

**Table 1:** Biopsy locations in primary and recurrent disease.

Location		first diagnosisn (%)	recurrencen (%)	totaln (%)
**lobar**	*frontal*	35 (17.0)	35 (17.0)	70 (34.0)
	*temporal*	13 (6.3)	8 (3.9)	21 (10.2)
	*parietal*	17 (8.3)	15 (7.3)	32 (15.5)
	*occipital*	3 (1.5)	9 (4.4)	12 (5.8)
	*central*	11 (5.4)	13 (6.3)	24 (11.7)
**deep seated**	*insular*	1 (0.5)	6 (2.9)	7 (3.4)
	*thalamic*	1 (0.5)	0 (0.0)	1 (0.5)
	*pineal*	3 (1.5)	0 (0.0)	3 (1.5)
	*cerebellar*	24 (11.7)	4 (1.9)	28 (13.6)
**brainstem**	*mesencephalon*	2 (1.0)	0 (0.0)	2 (1.0)
	*pons*	4 (1.9)	2 (1.0)	6 (2.9)
**total**		**114 (55.3)**	**92 (44.7)**	**206 (100)**

**Table 2** lists all primary tumor entities of the BM. Most frequent was lung cancer (39.8%), followed by breast cancer (9.2%) and malignant melanoma (7.3%).

**Table 2:** Listing of systemic tumor diseases.

Primary tumor entity	n (%)
Lung cancer	82 (39.8)
Breast cancer	19 (9.2)
Malignant melanoma	15 (7.3)
Cancer of unknown primary (CUP)	9 (4.4)
Gastrointestinal cancer	9 (4.4)
Squamous cell carcinoma	6 (2.9)
Thyroid cancer	5 (2.4)
Renal cell carcinoma	5 (2.4)
Other primary tumors	4 (1.9)
Gynecological tumor	3 (1.5)
Prostate cancer	3 (1.5)
Pseudoprogression	46 (22.3)
	Lung cancer 22 (47.8) Malignant melanoma 11 (23.9) Breast cancer 6 (13.0) Squamous cell carcinoma 3 (0.7) Other primary tumors 3 (0.7) Renal cell carcinoma 1 (0.2)
**Total**	**206 (100)**

In 114 out of 206 (55%) patients, BM were newly diagnosed. This included 45 cases with new-onset neurological symptoms and a first diagnosis of metastatic disease. In 64 patients, BM from known cancer diagnosis was confirmed. In 5 exceptional cases, the histologic examination revealed a metastatic origin different from the prior established cancer diagnosis.

In 92 (45% of) cases, STX was performed because of suspected tumor recurrence. As recommended by the interdisciplinary tumor board, additional [^18^F]-FET-PET imaging was available in 38 of these patients to rule out pseudoprogression/radionecrosis (**Table 3**). In this patient population, [^18^F] FET PET was indicative of tumor recurrence in 28 cases (subsequently confirmed histologically in 14 patients), while pseudoprogression/radionecrosis was noted in 10 cases (2 with histology showing tumor recurrence). This resulted in a sensitivity of 88% and specificity of 36% for [^18^F] FET PET to detect malignant progression, as well as a sensitivity of 80% and a specificity 88% of [^18^F] FET PET to determine cerebral reactive changes.

**Table 3:** Results of [^18^F] FET PET and stereotactic biopsy in suspected recurrences.

Histology of biopsy specimen	Tumor	Reactive Changes	Total
[^18^F]FET PET suggestive of tumor	14	14	16
[^18^F]FET PET suggestive of reactive changes	2	8	22
Total	28	10	38

Overall, neuropathological evaluation confirmed recurrent BM in 46 patients. These patients underwent additional treatment. In the other 46 patients, the biopsies showed only reactive changes consistent with pseudoprogression/radionecrosis. These latter patients were last pretreated with radiosurgery (n=19), fractionated stereotactic irradiation (N=8), interstitial brachytherapy (N=4) or systemic treatment (N=15), respectively. The median time between last treatment and occurrence of pseudoprogression/radionecrosis was 12 months (range, 3-112 months) and differed significantly from patients with proven tumor progression (median 7 months; Log-rank: HR 2.61; 95% CI of ratio 1.6-4.24; p<0.0001). Patients with pseudoprogression underwent close clinical and imaging follow-up, which ultimately confirmed reactive changes without active tumor activity in all these patients.

### Molecular analyses

Depending on the type of cancer confirmed histologically, certain biomarkers (all listed in **Table 4**) were requested by the interdisciplinary tumor board to establish the diagnosis and guide further therapeutic decisions.

**Table 4:** Molecular markers analysed among different tumor entities.

Primary tumor	Molecular marker	Positiven (%)	Negativen (%)	Inconclusiven (%)	Examinedn (%)Examined/Total (%)
Breast cancer	Her2/neu	8 (72.7)	2 (18.2)	1 (9.1)	11 (100.0) 11/19 (57.9)
	Estrogen receptor (ER)	13 (92.9)	1 (7.1)	0 (0.0)	14 (100.0) 14/19 (73.7)
	Progesteron receptor (PR)	6 (85.7)	1 (14.3)	0 (0.0)	7 (100.0) 7/19 (36.8)
Lung cancer	ALK	2 (6.3)	29 (90.6)	1 (3.1)	32 (100.0) 32/82 (39.0)
	ROS1	0 (0.0)	13 (86.7)	2 (13.3)	15 (100.0) 15/82 (18.3)
	EGFR	10 (31.3)	21 (65.6)	1 (3.1)	32 (100.0) 32/82 (39.0)
	PD-L1	9 (45.0)	11 (55.0)	0 (0.0)	20 (100.0) 20/82 (24.4)
Malignant melanoma	BRAF	7 (53.8)	4 (30.8)	2 (15.4)	13 (100.0) 13/15 (86.7)

For lung cancer metastases, ALK-protein and EGF-receptor (EGFR) were analyzed most frequently, with 31 conclusive cases out of 32 analyzed (97%). Furthermore, the PD-L1 surface protein was conclusively evaluated in 20/20 (100%) and the ROS1-protein in 13/15 cases (87%). In the 19 cases with breast cancer metastases, the estrogen-receptor (ER) was conclusively analyzed in 14/14 cases (100%), the progesteron-receptor in 7/7 cases (100%), and Her2/neu in 10/11 cases (91%). For patients with a malignant melanoma, molecular analysis was requested for the BRAF-gen in 13 cases and conclusive in 11 (85%). In total, the specific molecular genetic analysis was conclusive in 137 out of 144 cases (95%). Next-generation sequencing revealed 12 (67%) conclusive results in a small subgroup of 18 analyzed cases.

In addition, the molecular genetic signature of BM could be compared with the original tumor signature of 9 breast cancer patients regarding Her2/neu, ER and PR expression. From this group, 4 patients had an identical molecular signature, 3 had a partially matched signature, while in 2 cases a molecular signature different from the primary site was identified.

### Periprocedural complications

In 136 out of 206 cases (66%), a regular postoperative CT was performed. Minimal, clinically asymptomatic hemorrhages were visible in 59 postoperative CT scans (29%). Local hemorrhages with mild clinical symptoms occurred in 10 cases (4.9%). A space-occupying bleeding event was observed in one patient, which was successfully managed conservatively (**Table 5**). Overall, eloquent/deep-seated tumor location was not associated with an increased risk of bleeding.

**Table 5:** Complications according postoperative imaging.

CT (post-operative)	First diagnosisn (%)	Recurrencen (%)	Totaln (%)
no visible blood	77 (37.4)	59 (28.6)	136 (66.0)
Minimal hemorrhage	30 (14.6)	29 (14.1)	59 (28.6)
Local hemorrhage	6 (2.9)	4 (1.9)	10 (4.9)
Space-occupying hemorrhage	1 (0.5)	0 (0.0)	1 (0.5)
**Total**	**114 (55.3)**	**92 (44.7)**	**206 (100.0)**

A summary of complications according the CTCAE classification is provided in **Table 6**. Five (2.4%) patients reported mild symptoms (CTCAE grade 1) such as headaches, nausea, dizziness and rashes caused by perioperative antibiotics. CTCAE grade 2 complications were noted in two cases (1.0%), including one case of higher blood loss in need of transfusion most likely due to puncture of an intraosseous vein, and one case of perioperative atrial fibrillation. Severe symptoms (CTCAE grade 3) developed in 6 cases (2.9%): a paresis occurred in 3 cases after the intervention, one patient additionally presented with aphasia, and one with a fall due to this deficit. Two cases presented with a decreased level of consciousness immediately after the procedure, which resolved without further intervention. In all cases, CT scans were unremarkable. One patient without a prior history of epilepsy experienced a new focal tonic-clonic seizure. Overall, no life-threatening complications (CTCAE grade 4) or mortalities (CTCAE grade 5) emerge across the entire cohort. All complications were transient and resolved during the inpatient stay.

**Table 6:** Clinical complications according severity.

CTCAE	First diagnosis n (%)	Recurrence n (%)	Total n (%)
0	110 (53.4)	83 (40.3)	193 (93.7)
1	1 (0.5)	4 (1.9)	5 (2.4)
2	0 (0.0)	2 (1.0)	2 (1.0)
3	3 (1.5)	3 (1.5)	6 (2.9)
**Total**	**114 (55.3)**	**92 (44.7)**	**206 (100.0)**

## Discussion

In this retrospective analysis from a high-volume comprehensive cancer center, we addressed the diagnostic value and peri-procedural risk of a highly standardized, advanced imaging-based stereotactic biopsy technique. Furthermore, we performed extended molecular-genetic analyses in a sub-cohort. Overall, the diagnostic accuracy of representative tissue samples was found to be high and the associated risk was low, even in highly eloquent locations such as the brain stem. The high diagnostic certainty of >98% definite neuropathological diagnoses (only 4 inconlusive cases among 234 biopsies for suspected metastases) and low peri-procedural risk of 2.6% for clinically relevant transient morbidity is in line with our previous results on the value of stereotactic biopsy in a large cohort of primary brain tumors (23), and differs from retrospective analyses by other groups studying the respective diagnostic yield (up to 11% inconclusive results) (29).

The low procedural risk and high diagnostic yield of the collected tumor tissue is realized due to the combination of two relevant factors. First, a spatially precise fusion of advanced high-resolution imaging data (including MR-angiography and PET) to the frame-based CT-scan. Second, a versatile, small-sample size optimized neuropathological evaluation integrating intraoperative smear-preparation for representative tissue selection. Because of the low bleeding rate, we have largely eliminated postoperative cranial CT scans from our clinical routine and limit it to the rare cases with diagnostic uncertainty to rule out a missed biopsy.

No comparison was made to frameless biopsy procedures. At our institution, the latter technique is usually applied only for superficial primarily dural lesions without significant involvement of adjacent brain tissue and for extended cortical-subcortical tissue cubes when vasculitis is suspected. There are no prospective studies addressing the different biopsy techniques in terms of diagnostic yield and associated risk profiles. However, retrospective studies have demonstrated that frameless biopsy also provides good diagnostic value with low procedural risk (30). Whether this is also the case for highly eloquently located lesions in the midbrain or brainstem has not been clearly shown. Indeed, eloquent location was associated with in increased risk of periprocedural morbidity in 284 cases undergoing frameless biopsy (31). In our clinical experience, this subgroup of patients is often referred to us for further evaluation from other university and/or tertiary centers. In our hands there is no obvious disadvantage in terms of time of operating theater occupancy and staff retention compared to frameless procedures (30): Our stereotaxy system (Brainlab^®^ Elements Stereotactic Planning) already enables target-point-accurate trajectory planning the day before, which is merely supplemented by the information from the intraoperative CT. The actual operating time is usually 20 minutes. A major advantage of frameless systems, however, lies in the prevention of intraoperative radiation exposure.

The intraoperative presence of the neuropathologist certainly contributed to the high quality of our result. Although the results of the smear preparations did not result in a second trajectory being performed, the neuropathologist can help to minimize the total number of serial biopsies needed by providing early feedback, thereby reducing the overall risk of the procedure (32). This could be of particular benefit in highly vascularized tumors and in the case of highly eloquent tumor localizations such as the brainstem.

The study population reflects the current challenges in patients with BM. In this large cohort of over 450 patients in 5 years, we demonstrate that approximately 50% were not amenable for surgical resection, but were referred for biopsy as part of a risk-adapted interdisciplinary treatment regimen. Of note, only BM patients referred to our neuro-oncology center due to diagnostic uncertainty were included in this study. In clinical routine, many BM patients with a limited number of small BMs in known primary tumors as well as those with miliary seeding are usually scheduled for radiosurgery, stereotactic fractionated protocols, or whole-brain irradiation without being discussed in an interdisciplinary tumor board. The majority of our study patients underwent stereotactic biopsy in the setting of newly diagnosed brain metastasis. In 40% of these patients, BM biopsy was recommended to diagnose the systemic tumor because systemic biopsy was deemed either technically impossible or too risky. Remarkably, in a small subset of patients with newly diagnosed suspected brain metastasis (5/114, 4.4%), a previously unknown second tumor was detected.

After BM treatment, routine follow-up imaging is recommended in short intervals to readily detect tumor progression and to re-adjust treatment recommendations accordingly. However, the differentiation of tumor relapse from pseudoprogression/radionecrosis still represents a major challenge in BM. Standardized MRI as well as [^18^F] FET PET is routinely performed at our institution according to current RANO guidelines (20, 21). However, the diagnostic certainty of [^18^F] FET PET outlined in this study (sensitivity 87.5%, specificity 36.4% to detect malignant progression) is not sufficient to guide therapy decisions, so that the indication for tissue diagnosis has to be confirmed. In fact, reactive alterations without significant tumor cell content were observed in a striking 50% of patients and pseudoprogression could be confirmed due to the subsequent clinical course of disease in all these cases. The rate of reactive alterations may further increase if treatment approaches combining radiotherapy and immunotherapy are applied. However, this combination was rarely administered in this series, and as a result no such analysis could be performed. In our neuropathological diagnosis, the transition from reactive changes in the sense of a pseudoprogress to (symptomatic) radiation necrosis appears to be fluid. In the absence of clear neuropathological differentiation criteria, the interpretation often depends additionally on the clinical appearance and the image morphological findings and remains an individual decision. High numbers of radionecrosis, however, were reported in a case series of 2,200 BM patients treated with radiosurgery (33). Follow-up investigation confirmed a recurrence in 203 cases (46%), radionecrosis in 118 cases (27%), both recurrence and radionecrosis in 30 cases (6.8%), and 90 patients (20%) displayed inconclusive results. An even higher number of 69% histologically confirmed cases of radionecrosis were reported in 35 BM after radiosurgery (34). Therefore, STX as a minimal-invasive tissue sampling procedure for accurate tissue diagnosis will certainly gain increasing relevance in the era of precision medicine for BM (35–37).

The evolving landscape of effective targeted therapies has significantly altered the management paradigm of BMs (7, 38, 39). For example, targeted therapies have established intracranial activity in patients with Her2-positive breast cancer BM (10, 11), ALK-rearranged (12, 13) or EGFR-mutated NSCLC BM (40) and for BRAF V600E mutated melanoma BM (41). For subgroups of asymptomatic patients, targeted systemic therapy as monotherapy even represents a first-line consideration (41–43). Notably, tumor-dependent discrepancies can arise between the actionable mutational profile of the primary tumor and the respective BM (17). Strikingly, approximately 50% of brain metastases can harbor clinically relevant mutations that are not present in the primary tumor, indicating significant clonal heterogeneity across the various geographic regions of the tumor (44). Therefore, tissue-based analyses of BM are not only important to understand the pathogenesis of tumorigenesis, but are essential in guiding therapeutic concepts. Discordance in regards to EGFR status between brain metastases and matched NSCLC samples has been reported in 0–33% of cases, whereas the discordance rate for ALK rearrangements lies in the range of 0–13% (8). For breast cancer BM, a discordance rate of 14% for Her2 and 29% for ER/PR has been reported (45). Discordant molecular profiles were also observed in a small subgroup of breast cancer patients in this case series. Such discrepancies indicate a dynamic, clonal evolution of the spreading disease and has important implications for combinatorial treatment approaches (46). In this context, a safe and simple way to diagnose and longitudinally evaluate BM is of increasing clinical relevance.

In summary, the high diagnostic yield and low complication rate supports an important role for minimal-invasive biopsy procedures in risk-adapted management algorithms for BM. Since it is still an invasive intervention, a reasonable and cautious assessment of the individual indication and risk-benefit profile is clearly demanded. However, due to increasingly specialized teams and interdisciplinary cooperation, a high-quality standard of this procedure can be maintained. While other diagnostic methods, such as liquid biopsy, represent a less invasive examination method, they are less-researched, still of experimental nature in most cases, and do not have the same informational value as stereotactic biopsy (47, 48).

Our study has several important limitations. Due to the retrospective study design, several relevant questions such as the significance of [18F] FET-PET, timing of biopsy, and longitudinal treatment data, remain unanswered and warrant future systematic study. Important information concerning the intraoperative interaction between the stereotactic neurosurgeon and treating neuropathologist regarding the number and use (for smear preparation vs. final neuropathologic assessment or molecular genetic analysis) of serial tissue samples cannot be objectively recorded. In addition, no qualitative comparison can be made with other biopsy techniques, such as frameless procedures. In our study, the result of neuropathologic examination was the gold standard and the basis for any management decision in individual cases. Although in all cases the further clinical course supported a correct assessment, clinical misjudgment based on neuropathologic diagnosis cannot be excluded with absolute certainty.

In conclusion, image-guided stereotactic biopsy represents a valid and safe tool for diagnosis and even molecular characterization of BM. The precise identification of the molecular signatures of BM can guide the appropriate choice of targeted therapies, heralding a new era of precision medicine in the treatment of primary and recurrent brain metastases.

## Data availability statement

The datasets presented in this article are not readily available because of national and institutional laws to protect patient confidentiality. Requests to access the datasets should be directed to the Center for Neuropathology and Prion Research of the University Hospital of Munich.

## Ethics statement

Ethical approval for this analysis was obtained from the ethics committee of the Ludwigs-Maximilians University Hospital (project number 22-0476). Patients provided informed written consent to allow for anonymous or pseudonymous data handling.

## Author contributions

J-CT, SQ, LB and NT contributed to conception and design of the study. AD, SK, SQ, and NT organized the database, evaluated the clinical courses and performed image analyses. AD and SK carried out the statistical analyses. AD, SQ, SK, JW, J-CT, LB, and NT wrote the manuscript. MD, NA, RF, MN, and RE edited the manuscript. All authors contributed to manuscript revision, read and approved the submitted version.
